# A structural equation model linking health literacy, self-efficacy, and quality of life in patients with polycystic ovary syndrome

**DOI:** 10.1186/s12905-023-02223-4

**Published:** 2023-03-10

**Authors:** Yunmei Guo, Ying Liu, Rui Ding, Xin Yan, Huiwen Tan, Yousha Wang, Xueting Wang, LianHong Wang

**Affiliations:** 1 Nursing Department, Affiliated Hospital of Zunyi Medical University, Zunyi, China; 2 Nursing College, Zunyi Medical University, Zunyi, China

**Keywords:** Health literacy, Structural equation model, Quality of life, Self-efficacy

## Abstract

**Background:**

Health literacy is a crucial factor that affects health outcomes. Understanding the current status of health literacy among patients with polycystic ovary syndrome (PCOS) is the basis for helping patients better manage risk factors and improve their health outcomes. This study aimed to explore the status of and factors influencing health literacy in patients with PCOS, and to validate the pathway between health literacy, quality of life, and self-efficacy for these patients.

**Methods:**

A cross-sectional study was conducted using a convenience sample of 300 patients with PCOS in the gynecology outpatient clinic of a tertiary hospital in Zunyi from March to September 2022. Data on health literacy, demographic features, quality of life, and self-efficacy were collected. Multiple stepwise linear regression was conducted to assess the risk factors associated with health literacy for the study participants. A structural equation model was used to construct and validate the pathways.

**Results:**

Most participants exhibited low health literacy (3.61 ± 0.72), and only 25.70% had adequate health literacy. Multiple regression analysis revealed that the main factors associated with health literacy among participants included Body Mass Index (BMI) (B = −0.95, p < 0.01), education (B = 3.44, p < 0.01), duration of PCOS (B = 4.66, p < 0.01), quality of life (B = 0.25, p < 0.01), and self-efficacy (B = 0.76, p < 0.01). Multiple fit values indicated that the model fit the data effectively. The direct effect of health literacy on self-efficacy and quality of life was 0.06 and 0.32, respectively. The indirect effect of health literacy on quality of life was −0.053, and the total effect of health literacy on quality of life was 0.265.

**Conclusions:**

Health literacy was low among patients with PCOS. Healthcare providers should pay more attention to health literacy and to developing the corresponding intervention strategies urgently needed to improve the quality of life and health behavior of patients with PCOS.

## Background

Polycystic ovary syndrome (PCOS) is a common endocrine disease in women of reproductive age with a prevalence of 4%–22.5% [1]. PCOS is a multisystem disease characterized by anovulation, hyperandrogenism, and polycystic ovarian morphology [2]. To date, the cause of PCOS remains unclear. PCOS may increase the risk of type 2 diabetes, cardiovascular disease, endometrial cancer, metabolic syndrome, and pregnancy complications [2, 3]. Furthermore, PCOS can also significantly negatively impact psychological function [4]; the complex nature of the disease and its associated complications can diminish the quality of life (QOL) and lead to decreased emotional well-being [5]. Treatment for PCOS includes pharmacological options, surgical options, and life management. Although life management is recommended as a first-line treatment [6], maintaining a life management approach is a common challenge.

A previous study suggests that the challenges and barriers to implementing life management in patients with PCOS may be related to health literacy (HL) [7]. The World Health Organization defines HL as cognitive and social skills that determine an individuals' motivation and ability to understand and use information in a way that promotes and maintains health [8]. Having an adequate level of HL allows individuals to achieve the knowledge, skills, and confidence needed to improve personal health through lifestyle changes and to promote public health. Moreover, low or inadequate HL places a heavy burden on healthcare systems as it is associated with many poor health indicators, such as increased hospitalization and emergency department use, poor health outcomes, and higher mortality rates [10]. However, numerous studies [11, 12] have demonstrated that improving HL can constitute an essential breakthrough in promoting positive changes in health behavior. Unfortunately, only two articles [7, 13] have reported data related to HL in patients with PCOS and, thus far, the status of HL among patients with PCOS in China has not been investigated. Furthermore, the factors that affect HL have not yet been explored.

The evidence suggest that women with PCOS have poorer QOL compared with women with other chronic conditions, such as arthritis, back pain, and diabetes [14]. Previous studies have demonstrated that patients with low HL may be less concerned about their health status and therefore engage in unhealthy behavioral patterns that lead to a reduced QOL [15]. Similar studies have also suggested that HL can influence patients' QOL through self-efficacy (SE) [16–18]. SE is an individual’s belief in their ability to plan and execute a specific course of action [19]. SE management programs have been widely used in treating chronic diseases because they can improve functioning and health status [20]. The current study aims to affirm the role of SE in modulating psychological status and life management in PCOS [21, 22]. However, existing research on the relationship between SE and QOL [21, 22] has only identified a direct relationship through regression or correlation, and no study has explored this mediating relationship for patients with PCOS using structural equation modeling (SEM). SEM is a multivariate statistical framework used to model the complex relationships between directly and indirectly observed (latent) variables [23]. SE in PCOS patients may be a potential mediator, that is, an effective way to enhance HL and improve QoL. Therefore, it is necessary to use SEM to explore how HL can improve patients' QOL through SE.

To our knowledge, no previous study has investigated the status and associated factors of HL and validated the direct and indirect association between HL and QOL using SEM among patients with PCOS. To address this limited evidence, we conducted a cross-sectional study to investigate the status and influencing factors of HL in patients with PCOS and to validate the relationship between HL, SE, and QOL.

## Methods

### Design and data collection

A cross-sectional study was conducted in Zunyi between March and September 2022 among patients with PCOS attending a gynecology clinic. Patients were included if they met the following criteria: between 18–45 years of age with two of the following Rotterdam Criteria: a) hyperandrogenism, b) ovulatory dysfunction, and c) polycystic ovaries. Patients who were unable to read and understand the provided questionnaire, could not use a smartphone, or refused to sign the informed consent form were excluded from the study.

Three researchers conducted face-to-face data collection. After recruiting participants according to the inclusion criteria, the nature of the study, purpose, and investigation procedure were explained to them. All participants signed an informed consent form before participating in the study. Patients were instructed to complete a questionnaire using a smartphone scan code. While the participants were completing the questionnaire, one of the researchers checked the questionnaire filling status. To reduce the generation of invalid questionnaires, researchers checked and confirmed incorrect or incomplete responses in real time.

### Sample size calculation

We calculated the sample size using event-per-variable (EPV), assuming that p represents the prevalence of PCOS and K represents the number of predictors. Based on the above assumptions and the formula N = EPV × K/p (k = 4, p = 0.15), only an EPV of 10 or above is considered robust. According to the above formula, the sample size was 267, and the final sample size was 307, considering a 15% sample loss rate.

### Measures

#### Demographic questionnaire

A checklist included questions on the following demographic features: waist circumference, age, height, weight, years of illness, place of residence, marital status, education level, body mass index (BMI), and occupation.

#### Health literacy scale

In this study, the Chinese version of the Health Literacy Management Scale (HeLMS) was used to evaluate HL of patients [24, 25]. This questionnaire consists of 24 items in four dimensions: communication and interaction ability (9 items with a total score of 45 points), information acquisition ability (9 items with a total score of 45 points), willingness to improve health (4 items with a total score of 20 points), and willingness to financially support health (2 items with a total score of 10 points). Each item is rated on a 5-point Likert scale ranging from 1 (very difficult) to 5 (not at all difficult), with higher scores indicating a more advanced level of HL. The total score is 120 points. An average score of < 4 on all dimensions of this scale was considered as inadequate or low level of HL. The internal consistency of the HeLMS scale was good, with a Cronbach's α of 0.874. Dimension mean scores = total score per dimension/total participants; score per dimension item = total score per dimension/number of dimension items; item mean scores = total score per dimension item/total participants; and possessing rate = total number of items with a score of less than four points per dimension/total participants.

#### Quality of life [26]

QOL was calculated using the Short Form 36 (SF-36) scale. It comprises eight subscales: general health, bodily pain, physical functioning, vitality, role limitations due to physical problems, mental health, role limitations due to emotional problems, and social function. A higher score indicates a better QOL and the scores range from 0 to 100. The internal consistency of the SF-36 scale was good, with a Cronbach’s α of 0.89.

#### Self-efficacy scale

SE was assessed using the General Self-Efficacy Scale, which consists of 10 items. A four-point Likert scale was used to answer each question, ranging from 1 (not at all true) to 4 (exactly true). Total scores range from 10 to 40, with higher scores indicating better SE [27].

### Ethical considerations

This study was approved by the Ethics Committee of the Affiliated Hospital of the Zunyi Medical University (No. KLLY-2020-134). Informed consent was obtained from all participants, and procedures were conducted according to the Declaration of Helsinki. All participants had the right to withdraw at any time without any adverse effect on the clinical work.

### Data analysis

SPSS18.0 was used to analyze the data. Descriptive data is expressed as frequencies and mean ± standard deviation. Demographic differences in HL, SE, and QOL were analyzed using an independent samples t-test and ANOVA. Correlations between HL, SE, and QOL were analyzed using Pearson’s correlation analysis. Multiple stepwise linear regression was performed to examine the risk factors for HL. P values less than 0.05 indicated a significant difference. AMOS 26.0 was used to model the structural equations, with HL, QOL, and SE as latent variables and the corresponding entries set as observed variables. The models were continuously improved and re-estimated, and the most appropriate model was selected.

## Results

Seven patients declined to participate in this study because of time pressure (N = 4) and too many questionnaire items (N = 3). Ultimately, 300 patients with PCOS were included in the study. Table 1 presents participants' general demographic information. The average age was 24.78 ± 4.21 years, the average BMI was 24.69 ± 3.43, and the average waist circumference was 81.83 ± 7.84. The majority of women had been educated beyond middle school (n = 233, 77.70%), 52.70% of the patients were married, and 52.30% reported that they wished to have children. Further, 47.70% reported having PCOS for less than 1 year and 33.30% reported having PCOS for 1–3 years. The mean scores for HL, SE, and QOL of the patients were 87.20 (SD = 17.20), 26.55 (SD = 6.56), and 56.11(SD = 10.55), respectively.

**Table 1:** Demographic characteristics of participants (N = 300)

Variable	Categories	Mean (SD)	Frequency (N)	Percentage (%)
Age		24.78 ± 4.21		
BMI		24.69 ± 3.43		
WC		81.83 ± 7.84		
Residence	City		158	52.70
	Countryside		142	47.30
Marital status	Single		136	45.30
	Married		158	52.70
	Widowed/divorced		6	2.00
Ethnic group	Han-Nationality		251	83.70
	Ethnic minority		49	16.30
Education	Elementary		67	22.30
	Middle school		58	19.30
	High school		54	18.00
	College		121	40.30
Occupation	Employed		99	33.00
	Unemployed		57	19.00
	Student		63	21.00
	Other		81	27.00
Duration of PCOS	< 1 year		143	47.70
	1–3 years		100	33.30
	4–6 years		36	12.00
	> 7 years		21	7.00
Desire for pregnancy	Yes		157	52.30
	No		143	47.70
Self-efficacy		26.55 ± 6.56		
Health literacy		87.20 ± 17.20		
Quality of life		56.11 ± 10.55		

*BMI* body mass index, *WC* waist circumference

Table 2 shows the total HL scale scores and mean scores for each sub-dimension item. The average total HL was 87.20 ± 17.01 (mean: 3.61 ± 0.72). The total score for information acquisition ability was 32.47 ± 7.71 (mean: 3.67 ± 0.86), communication and interaction ability was 32.46 ± 7.47 (mean: 3.61 ± 0.83), willingness to improve health was 15.23 ± 3.72 (mean: 3.81 ± 0.92), and willingness to financially support health was 6.85 ± 2.28 (mean: 3.43 ± 1.14). Only 25.70% of patients had adequate health literacy.

**Table 2:** HL total and subscale mean scores (N = 300) of patients with PCOS

Item	Dimension mean scores	Item mean scores	Highest and lowest obtainable score	Possessing rate (%)
Information acquisition ability	32.47 ± 7.71	3.67 ± 0.86	9–45	32.70
Communication and interaction ability	32.46 ± 7.47	3.61 ± 0.83	9–45	25.00
Willingness to improve health	15.23 ± 3.72	3.81 ± 0.92	5–20	33.00
Willingness to support financially	6.85 ± 2.28	3.43 ± 1.14	2–10	18.70
Total mean scores	87.20 ± 17.01	3.61 ± 0.72	25–120	25.70

As shown in Table 3, there were significant differences in the scores of HL and SE between married and unmarried and widowed/divorced patients (p < 0.01). There were significant differences in HL, QOL, and SE among patients with different levels of education (p < 0.001) and with different PCOS durations (p < 0.001). Additionally, as shown in Table 4, HL was positively and statistically associated with QOL and SE (p < 0.01). This study showed that HL was negatively associated with BMI.

**Table 3:** Demographic characteristics and their associations with health literacy, self efficacy, and quality of life(N = 300)

Variable	Categories	Frequency (N)	HL (mean ± SD)	QOL (mean ± SD)	SE (mean ± SD)
Residence	city	158	86.11 ± 16.18	50.55 ± 10.85	26.86 ± 6.68
	Countryside	142	88.42 ± 17.87	52.78 ± 10.11	26.19 ± 6.44
	P value		0.173	0.093	0.328
Marital status	Single	136	84.47 ± 17.94	51.63 ± 11.05	25.87 ± 6.23
	Married	158	88.96 ± 15.81	51.31 ± 10.16	26.81 ± 6.18
	Widowed/divorced	6	102.66 ± 13.00	58.65 ± 7.33	34.67 ± 15.23
	P value		**0.006**	0.247	**0.004**
Ethnic group	Han-Nationality	251	87.48 ± 17.19	51.78 ± 10.37	26.63 ± 6.44
	Ethnic minority	49	85.75 ± 16.09	50.71 ± 11.47	26.14 ± 7.22
	P value		0.694	0.15	0.33
education	Elementary	67	74.82 ± 15.23	47.98 ± 10.63	24.31 ± 6.19
	Middle school	56	83.08 ± 17.58	51.84 ± 11.01	24.38 ± 5.37
	High school	54	93.38 ± 13.46	52.96 ± 9.98	27.57 ± 5.35
	College	121	93.26 ± 14.78	52.89 ± 10.17	28.36 ± 7.15
	P value		**0.000**	**0.014**	**0.000**
Occupation	Employed	99	84.78 ± 16.83	50.56 ± 10.84	26.55 ± 6.24
	Unemployed	57	84.47 ± 17.09	50.64 ± 11.52	25.17 ± 6.52
	Student	63	89.77 ± 15.97	53.14 ± 9.92	26.98 ± 5.81
	Other	81	90.06 ± 17.50	52.36 ± 9.92	27.16 ± 7.45
	P value		0.065	0.363	0.325
Duration of PCOS	< 1 year	143	80.04 ± 15.49	49.57 ± 10.66	24.90 ± 6.18
	1–3 years	100	89.21 ± 15.56	51.31 ± 10.53	27.00 ± 5.93
	4–6 years	36	102.36 ± 12.65	56.88 ± 8.68	29.11 ± 5.73
	> 7 years	21	100.38 ± 12.58	57.81 ± 7.41	31.19 ± 9.33
	P value		**0.000**	**0.000**	**0.000**
Desire for pregnancy	Yes	157	87.29 ± 17.49	51.86 ± 10.18	26.75 ± 6.90
	no	143	87.09 ± 16.53	51.32 ± 10.96	26.32 ± 6.19
	P value		0.50	0.171	0.618

The bold definition is statistically significant

*HL* health literacy, *SE* self-efficacy, *QOL* quality of life

**Table 4:** Associations and differences of HL mean scores with demographic variables (N = 300)

Variable	information acquisition ability	communication and interaction ability	willingness to improve health	willingness to support financially	Total score
Age	−0.086	−0.069	−0.005	−0.08	−0.084
BMI	−0.312**	−0.222**	−0.195**	−0.181**	−0.303**
WC	−0.156**	−0.042	−0.069	−0.067	−0.112
SE	0.383**	0.422**	0.361**	0.328**	0.484**
PF	0.405**	0.487**	0.245**	0.292**	0.494**
RP	0.202**	0.177**	0.230**	0.240**	0.246**
BP	−0.065	−0.111	−0.137**	−0.164**	−0.130**
GH	0.187**	0.180**	0.062	0.094	0.189**
VT	0.073	0.135**	0.098	0.083	0.128**
SF	−0.043	−0.083	−0.029	−0.033	−0.066
RE	0.176**	0.204**	0.174**	0.203**	0.235**
MH	0.100	0.168**	0.148**	0.019	0.158**
QOL	0.28**	0.336**	0.211**	0.161**	0.346**

**p < 0.05; *BMI* body mass index; *WC* Waist circumference; *HL* health literacy, *SE* self-efficacy; *QOL* quality of life, *GH* general health, *BP* bodily pain, *PF* physical functioning, *VT *vitality, *RP* role limitations due to physical problems, *MH* mental health, *RE* role limitations due to emotional problems, *SF* social function

Table 5 shows the results of the stepwise multiple linear regression analysis, which revealed that factors including BMI (B = −0.95, p < 0.01), education (B = 3.44, p < 0.01), duration of PCOS (B = 4.66, p < 0.01), QOL(B = 0.25, p < 0.001), and SE (B = 0.76, p < 0.01) were associated with HL. The model showed 47.40% variance shared between the dependent and independent variables (R2 = 47.40, F = 52.97, p < 0.001).

**Table 5:** Multivariate analysis (stepwise) of predictors for HL(N = 300)

Model	Variable	Unstandardised coefficients		Standardised coefficients	t	sign	95.0% CI for B		R2	F	P
		B	SE	Beta			Lower bound	Upper bound			
	BMI	−0.95	0.21	−0.19	−4.43	0.00	−1.37	−0.53	47.40	52.97	0.00
	Education	3.44	0.64	0.24	5.40	0.00	2.19	4.70			
	Duration of PCOS	4.66	0.86	0.25	5.43	0.00	2.97	6.35			
	QOL	0.25	0.07	0.16	3.52	0.00	0.11	0.39			
	SE	0.76	0.12	0.29	6.45	0.00	0.53	0.99			

*BMI* body mass index, *HL* health literacy, *SE* self-efficacy, *QOL* quality of life

**Fig. 1:**
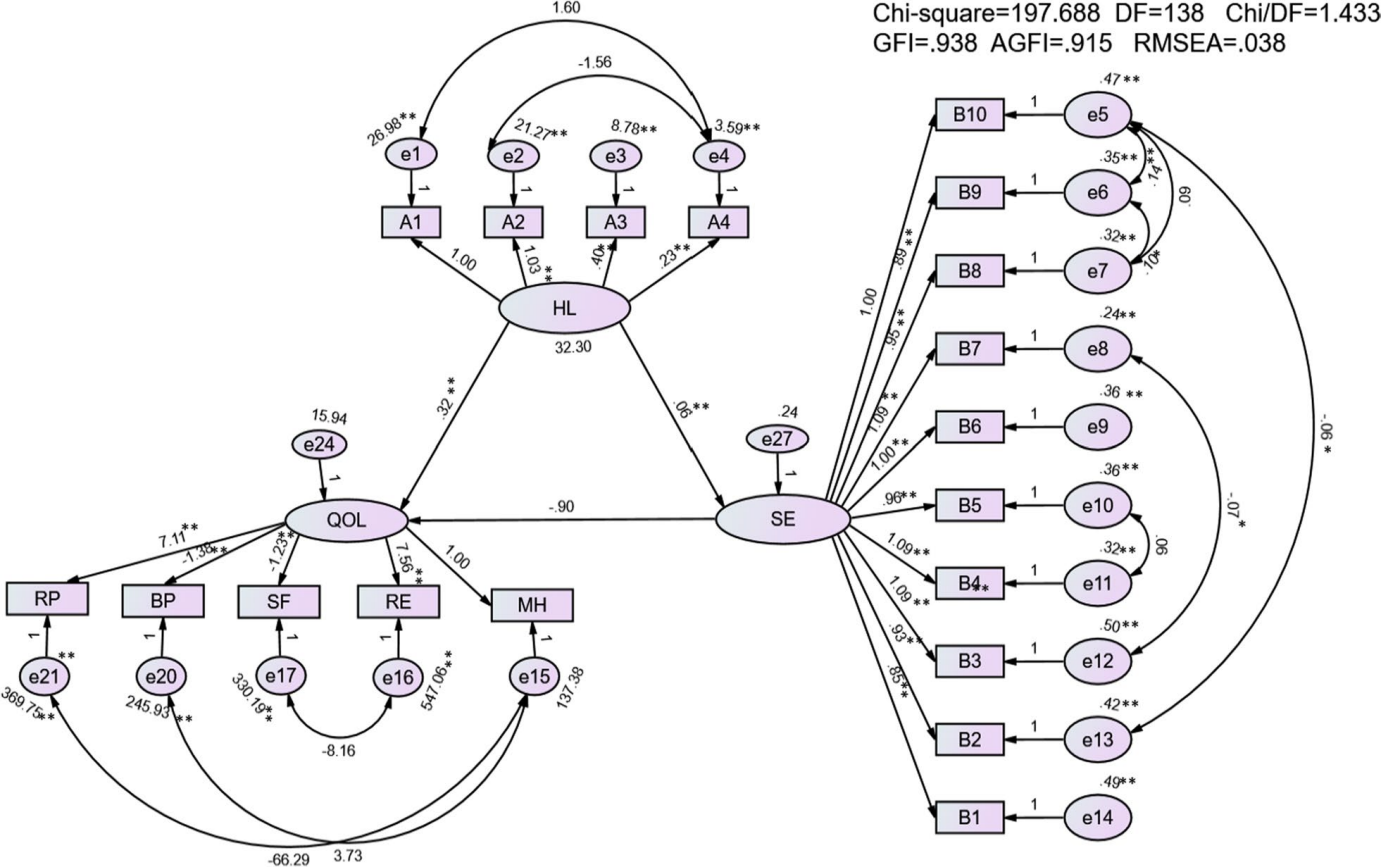
Final model and standardized pathway coefficients among health literacy, self efficacy, and quality of life. Note:*P < 0.05; **P < 0.01; HL = health literacy; SE = self-efficacy; QOL = quality of life; A1 = Information acquisition ability; A2 = Communication and interaction ability; A3 = Willingness to improve health; A4 = Willingness to support financially; RP = Role limitations due to physical problems; BP = bodily pain; SF = social function; RE = role limitations due to emotional problems; MH = mental health; B1 = If I do my best, I can always solve the problem; B2 = Even if others are against me, I still have a way to get what I want.; B3 = It is easy for me to stick to my ideals and achieve my goals; B4 = I am confident that I can deal with anything that happens all of a sudden.; B5 = With my intelligence, I will be able to cope with unexpected situations.; B6 = If I do what I have to do, I will be able to solve most of the problems.; B7 = I can face difficulties calmly because I believe in my ability to deal with problems.; B8 = . When faced with a difficult problem, I can usually find several solutions; B9 = When there is trouble, I can usually think of some ways to deal with it; B10 = No matter what happens to me, I can handle it

The final model fit indicated that the χ2/degree of freedom was 1.433, goodness of fit index was 0.938, adjusted goodness to fit was 0.915, comparative fit index was 0.971, normed fit index was 0.912, Tucker-Lewis index was 0.965, incremental fit index was 0.972, and the root mean square error of approximation was 0.038 (Table 6). Further, SE mediated the influence of HL on QOL. The direct effect of HL on SE and QOL was 0.06 and 0.32, respectively. The indirect effect of HL on QOL was −0.053 and the total effect of HL on QOL was 0.265. These fit indices suggest that SEM better describes the pathway relationship between HL, SE, and QOL (Fig. 1).

**Table 6:** Model fit index

Variable	χ^2^	GFI	RMSEA	NFI	TLI	CFI	IFI	AIC
Fit index	197.688	0.938	0.038	0.912	0.965	0.971	0.972	401.23
Reference value		> 0.9	< 0.05	> 0.9	> 0.9	> 0.9	> 0.9	> 400

*GFI* goodness of fit index, *CFI* comparative fit index, *NFI* normed fit index, *TLI* =Tucker-Lewis index, *IFI* incremental fit index, *RMSEA* root mean square error of approximation, *AIC* Akaike Information Criterion

## Discussion

This study is the first to investigate the status of HL in patients with PCOS in China, and the findings showed that most patients (74.30%) had inadequate HL (87.20 ± 17.20), and only 25.70% had adequate HL. In addition, to the best of our knowledge, this is the first study to use SEM to construct and validate the pathway between HL, SE, and QOL in patients with PCOS. HL directly impacted QOL and SE in patients with PCOS. It was revealed that SE played a mediating role between HL and QOL.

In the current study, we found that patients with PCOS had low HL (3.61 ± 0.72), which is consistent with previous studies [13]. However, compared with other chronic diseases, the health literacy of PCOS patients is lower than that of hemodialysis patients and diabetes patients [28, 29]. Recent research has shown that the level of health literacy in the eastern coastal provinces of China is higher than that in the central and western provinces [30]. Considering that all the participants included in this study were from western China, the factor of geographical location may have led to low HL in patients. Furthermore, recent research has shown that doctors in China, Europe, and America lack knowledge regarding PCOS [31–34], and insufficient disease education in doctors may reduce the abilities for information acquisition and HL in patients. Finally, low health literacy in patients may be due to the fact that the clinical symptoms of PCOS do not endanger patients' lives and that there is lack of knowledge about the long-term complications of the disease [31, 32]. Therefore, future studies need to address how to strengthen the continuing education about PCOS for Chinese obstetricians and gynecologists, improve the quality of medical services, improve the health system at all levels, and use the internet and digital technology to solve the changing health literacy needs of individuals and communities. Furthermore, research is needed on how to formulate appropriate intervention measures according to local culture and to design policies to continuously improve the HL of patients in the western region.

This study showed that HL was positively associated with education and disease duration and negatively associated with BMI. First, it was found that higher educational levels in patients with PCOS were associated with better HL scores. This shows that education level is a significant factor in enhancing and improving HL. PCOS is the most common endocrine disease that requires long-term management [35], and adequate HL for patients with low education levels may enhance the effect of life management on patients' reproductive health and metabolism and promote the maintenance of healthy behaviors. Second, it was revealed that a higher duration of PCOS resulted in higher HL scores. A longer disease duration may increase the accessibility of health education and eventually enhance the skills required for disease knowledge and information. Finally, the negative relationships between HL scores and BMI were congruent with previous findings [18]. The negative correlation between HL and BMI suggests that patients with high HL have healthier lifestyles [36]. Moreover, previous studies have suggested that a higher BMI is associated with poor health behaviors in the life management of patients with PCOS [21], which may explain the relationship between a high BMI and low HL. Furthermore, chronic disease prevalence increases with increasing BMI [37, 38] and, since PCOS is a chronic disease and most patients are obese, this may also contribute to the negative relationship between HL and BMI.

In the current study, we discovered that the direct effects of HL on patients' QOL by SEM were congruent with previous findings [37, 38]. Previous studies have demonstrated that patients with low HL may be less concerned about their health status and therefore have unhealthy behavioral patterns that lead to a reduced QOL [15]. In addition, PCOS patients with low health literacy may have difficulty in fully understanding the instructions from healthcare providers. They may also have difficulty in maintaining life management, potentially resulting in suboptimal symptom management (e.g., metabolic and reproductive disorders), all of which may exacerbate treatment-related symptoms, resulting in a poorer QOL [7, 39]. This may explain the negative association between HL and QOL. Patients with higher SE may develop more strength, greater awareness, and intrinsic motivation, allowing them to persist in treatment and life maintenance. This study confirms that SE is a mediator between HL and QOL. Patients with PCOS that are deficient in HL may have lower SE in their daily lives, thereby reducing their QOL, a relationship that echoes previously proposed theoretical frameworks [40]. Finally, doctors often ignore the evaluation and management of declines in patients’ psychosocial status and QOL [31, 32]. Doctors' neglect of patients' QOL and lack of education may be potential reasons for the decline in patients' QOL.

The strength of our study is that it is the first to investigate HL status and confirm its associated risk factors. Furthermore, it is the first study to investigate the pathways between HL, SE, and QOL, while also confirming the direct and indirect effects of HL on QOL among patients with PCOS. However, this study has some limitations. First, patients were recruited using convenience sampling from the gynecology outpatient clinic of only one tertiary hospital in China. Second, data for this study were collected from patients through self-report. Therefore, the possibility of a response bias could not be eliminated. Third, owing to the limitations of cross-sectional studies, no causal relationship can be inferred; only correlations between variables can be analyzed. Finally, the HL scales used to investigate the patients in this study were not specific, which may have led to biased results.

## Conclusions

In summary, the study revealed that patients with PCOS had low HL; the factors affecting HL mainly included BMI, education, duration of PCOS, SE, and QOL. Moreover, our research confirmed the direct and indirect effects of HL on QOL using SEM. Therefore, future studies should focus on improving and promoting patients' HL and enhancing medical staff awareness to improve patients' HL. The results of this study fill a gap in the literature and provide new perspectives for further improving the QOL and behavior management of patients with PCOS.

## Data Availability

All the original data of this study are not uploaded to the database publicly, but you can contact the correspond and the first author to obtain the original data.

## Abbreviations

HL: Health literacy
QOL: Quality of life
SE: Self-efficacy
PCOS: Polycystic ovary syndrome
SEM: Structural equation modeling
BMI: Body mass index
EPV: Event-per-variable
HeLMS: Health Literacy Management Scale
